# Diagnostic test accuracy of screening tools for the detection of neurocognitive disorders in older adults post-trauma: A protocol for a systematic review

**DOI:** 10.12688/hrbopenres.13894.1

**Published:** 2024-06-04

**Authors:** Niamh A. Merriman, Mary E. Walsh, Niamh O'Regan, Marie Carrigan, Pamela Hickey, Louise Brent, Catherine Blake

**Affiliations:** 1 School of Public Health, Physiotherapy and Sports Science, University College Dublin, Dublin, Ireland; 2 University Hospital Waterford, Waterford, Ireland; 3 Health Information and Quality Authority, Dublin, Ireland; 4 National Office of Clinical Audit, Dublin, Ireland

**Keywords:** systematic review protocol, diagnostic accuracy, cognition, delirium, older adults

## Abstract

**Background:**

Neurocognitive disorders (NCDs), including delirium, cognitive impairment, or dementia are prevalent in up to 39% of older adults in acute care, particularly older trauma patients. Undiagnosed NCDs result in poor outcomes, such as increased incidence of depressive symptoms, longer length of stay, and mortality.

**Objective:**

This study aims to identify the diagnostic test accuracy of screening tools for the detection of NCDs in older trauma patients in acute settings.

**Design:**

Systematic review protocol.

**Literature search:**

Electronic databases (MEDLINE, Embase, CINAHL, PsycInfo, Cochrane Library) will be searched for journal articles. Search terms related to NCDs, delirium and cognitive screening tools, and diagnostic accuracy will be included.

**Study selection criteria:**

Cross-sectional, prospective, or retrospective cohort studies of adults aged ≥60 post-trauma, in an acute setting, will be included where the study aimed to validate a screening tool for detection of 1) delirium or 2) cognitive impairment, or dementia against a reference standard of a clinical decision, based on standardised diagnostic criteria or a validated tool.

**Data synthesis:**

Two review authors will conduct study selection, data extraction, and appraisal. Data will be extracted based on the Preferred Reporting Items for a Systematic Review and Meta-analysis of Diagnostic Test Accuracy Studies (PRISMA-DTA) checklist. Studies will be assessed for methodological quality by two independent review authors using the Quality Assessment of Diagnostic Accuracy Studies (QUADAS-2) tool. Narrative summaries will be generated describing risk of bias and concerns regarding applicability. Quantitative synthesis of study findings will be conducted.

**Conclusion:**

This systematic review will aim to identify screening tools with the best diagnostic accuracy for detection of 1) delirium and 2) cognitive impairment or dementia in adults aged ≥60 post-trauma in acute care settings. Results will inform clinical practice to enhance the probability of patients with NCDs receiving appropriate care and management.

**Registration:**

PROSPERO https://www.crd.york.ac.uk/prospero/display_record.php?ID=CRD42024518730 (11/03/2024).

## Introduction

Neurocognitive disorders (NCDs), such as delirium, cognitive impairment, or dementia are prevalent in 21% to 39% of older adults in acute care ^1, 2^. In general, older adults post-trauma (i.e., following physical trauma) in acute care have complex ongoing healthcare needs, with high prevalences of delirium and cognitive impairment ^3^, particularly in patients with hip fracture ^4, 5^. Trauma is defined as a physical injury of sudden onset and severity which requires immediate medical attention ^6^. Delirium is an acute and fluctuating NCD, characterised by impaired attention, altered awareness, and other cognitive and neuropsychiatric disturbances ^7^. Cognitive impairment is an NCD, characterised by a disruption to some cognitive function such as memory, though independence in functional abilities is preserved ^8, 9^. Dementia is an NCD in which there is deterioration in cognitive function beyond that which might be expected from the usual consequences of biological ageing and which affects a person’s ability to function independently ^10^. Despite the high prevalence of NCDs, 56% to 62% of cases are undiagnosed or undetected by healthcare professionals ^11, 12^. Undiagnosed NCDs can lead to poor outcomes, such as increased incidence of depressive symptoms, longer length of stay, and mortality ^13, 14^.

Over half of patients admitted to hospital following physical trauma are over 60 years old ^15^, with the majority of injuries resulting from low-energy falls of less than 2 metres ^15^. Other types of trauma include motor vehicle collisions, falls from more than 2 metres, and intentional injury ^3, 15^. Hip fracture is the most serious consequence of falls among adults ^16^, and older adults with hip fracture are at increased risk of NCDs. A systematic review of observational studies found that 19% of people with hip fracture meet formal diagnostic criteria for dementia, and 42% have cognitive impairment ^17^. It is expected that the number of people living with dementia will double worldwide to 75 million by 2030 and 131.5 million in 2050 ^18^. Therefore, the number of people with cognitive impairment or dementia and hip fracture will increase over the next 25 years ^19^. Older adults with cognitive impairment or dementia are likely to have a complex hip fracture care pathway, due to their greater risk of poor outcomes and need for person-centred, tailored support ^19^. The high incidence of delirium (up to 30%) among hip fracture patients impacts on cognitive screening ^5, 20, 21^. Delirium itself can result in increased length of stay, long-term cognitive and functional decline, increased risk of discharge to long-term care, higher mortality, and patient and carer distress. Although management of delirium is an essential facet of acute geriatric care, older adults with pre-existing cognitive impairment without delirium are at high risk of being undiagnosed ^22^.

Given the breadth of available cognition and delirium screening tools, the variability in recommended cut-off thresholds, and the adverse outcomes for older adults with NCDs post-trauma in acute care, it is timely to conduct a diagnostic accuracy review of screening tests for cognitive impairment and delirium in older adults. The findings will contribute greatly to clinical practice and policy making by identifying the 1) delirium and 2) cognitive/dementia screening tools with the best diagnostic test accuracy for use with older adults post-trauma in acute care to enhance the probability of patients with NCDs receiving appropriate care and management.

## Protocol

### Study design

A systematic review of studies that describe the accuracy of screening tools for 1) delirium and 2) cognitive impairment or dementia in older adults admitted to acute care following physical trauma compared with a clinical reference standard based on the Diagnostic and Statistical Manual of Mental Disorders (DSM) criteria (version IV or V) ^7, 23^, the International Classification of Diseases (ICD) ^24^, or a validated tool will be conducted. This protocol follows the PRISMA-P Reporting Guidelines ^25^ (see Extended data ^26^). The systematic review protocol was registered with the International Prospective Register of Systematic Reviews (PROSPERO) on 11 March 2024 ( https://www.crd.york.ac.uk/prospero/display_record.php?ID=CRD42024518730). If applicable, important protocol updates will be registered via PROSPERO. The review will be carried out and reported in accordance with two guidelines: the Standards for Reporting of Diagnostic Accuracy Studies (STARD 2015) ^27^ and the Preferred Reporting Items for a Systematic Review and Meta-analysis of Diagnostic Test Accuracy Studies (PRISMA-DTA) checklist ^28^.

### Selection criteria

Studies will be included if:

- Population consists of older adults aged 60 years or above or has median age above 70 years in an acute care setting following physical trauma as the main study group or as a clearly defined subgroup. Trauma is defined as a physical injury of sudden onset and severity which requires immediate medical attention ^6^.
- The study aims to validate a screening tool for detection of 1) delirium or 2) cognitive impairment, or dementia at any timepoint in acute care.
- The study aims to diagnose 1) delirium or 2) cognitive impairment or dementia using a clinical decision, based on standardised diagnostic criteria or a validated tool.
- The study design is a cross-sectional, prospective, or a retrospective cohort study. Control arms of randomised controlled trials will also be considered for inclusion where relevant.
- They are published in peer-reviewed journal articles.
- They are reported in any language.
- The study provides sufficient data to construct a two-by-two table.

Studies will be excluded if any of the following apply:

- The study focuses only on younger people (under 60 years).
- The population does not consist of physical trauma patients.
- Case reports or case-control design.
- Studies conducted in environments other than acute care (e.g., primary care, memory clinic, outpatient, rehabilitation settings, or community settings) will be excluded.

### Search strategy

A comprehensive search strategy will be developed in collaboration with a librarian and a search of electronic databases will be conducted, including MEDLINE (Ovid), Embase (Elsevier), PsycInfo (EBSCO), CINAHL (EBSCO), and the Cochrane Library (Wiley). Search terms related to neurocognitive disorders, delirium and cognitive screening tools, and diagnostic test accuracy ^29^ will be included. No date or language restrictions will be applied. Citation lists of previous systematic reviews and included studies will be hand-searched. See Extended data for full search strategy for all sources ^30^.

### Study selection

All citations identified from the search strategy will be exported to EndNote (Version X20) for reference management, where duplicates will be identified and removed. Using Covidence Systematic Review Software ^31^, two reviewers will independently review the titles and abstracts of the remaining citations to identify those for full-text review. The full texts will be obtained and independently evaluated by two reviewers, applying the defined inclusion and exclusion criteria. Where disagreements occur, discussions will be held to reach consensus and where necessary, a third reviewer will be involved. Citations excluded during the full-text review stage will be documented alongside the reasoning for their exclusion and included in the PRISMA flow diagram ^32^. Study authors will be contacted if further information is required to determine eligibility.

### Data extraction

Data extraction will be performed by one reviewer and checked for accuracy and omissions by a second. Where disagreements occur, discussions will be held to reach consensus and where necessary, a third reviewer will be involved. Data will be extracted on: 1) title, first author, year and country; ii) type of study; iii) setting; iv) study population; v) comorbid illness or illness severity, if reported; vi) patient demographics; vii) number of patients in total, that is a) n with delirium, n without delirium and b) n with mild cognitive impairment or dementia, n without mild cognitive impairment or dementia; viii) details of screening tool administration (timing, assessor, etc.) and the condition reference standard process; ix) details of screening tool implementation (acceptability, inter-rater reliability, training, etc.); x) test accuracy data, extracted to a two-by-two table (number of true positives, false positives, true negatives and false negatives); xi) statistics used, including adjustments made; xii) conclusion of study; and, xiii) comments on study quality.

### Risk of bias in individual studies

Risk of bias assessment will be conducted using the Quality Assessment of Diagnostic Accuracy Studies (QUADAS-2) tool ^33^. Narrative summaries will be provided describing risk of bias (high, low, or unclear) and concerns regarding applicability. All included studies will be appraised independently by two designated authors. Disagreements will be resolved through discussion and a third author will be involved if necessary.

### Data synthesis

Characteristics and results of all studies will be narratively synthesised and presented in tables. Quantitative synthesis of the findings from the included studies will be conducted. From the data extracted from the included studies, 2 × 2 tables will be constructed for presence or absence of delirium or dementia/cognitive impairment. Summary estimates of sensitivity and specificity with 95% confidence intervals will be calculated. Meta-analysis will be conducted if two studies reporting on the same screening instrument are included. A random effects bivariate meta-analysis of sensitivity and specificity will be conducted using Diagnostic Test Accuracy Meta-Analysis software, version v2.1.1 ( https://crsu.shinyapps.io/MetaDTA/) ^34, 35^. A sensitivity analysis will be performed on studies which were deemed to have an overall low risk of bias. Sub-group analyses will be conducted based on study population (i.e., post-hip fracture, post-surgical, other physical trauma) and on type of reference standard (i.e., standardised diagnostic criteria or a validated tool).

## Study status

Pilot searches were conducted in February 2024. Main searches were conducted in March 2024. Title and abstract screening and final study selection will be completed from March to July 2024. Data extraction and quality appraisal will be completed from August to October 2024.

## Conclusions

This systematic review will aim to identify, critically appraise, and synthesise the diagnostic accuracy of available screening tools for detection of 1) delirium and 2) cognitive impairment or dementia in adults aged ≥60 in acute care settings following physical trauma. The findings will inform clinical practice and policy making to enhance the probability of patients with NCDs receiving appropriate care and management.

## Data Availability

No data are associated with this article. Zenodo: Extended Data for ‘Diagnostic test accuracy of screening tools for the detection of neurocognitive disorders in older adults post-trauma: A protocol for a systematic review’. https://doi.org/10.5281/zenodo.10978778 ^30^. This project contains the following extended data: Full Search Strategy Data is available under the terms of Creative Commons Attribution 4.0 International (CC by 4.0) Zenodo: PRISMA-P Checklist for ‘Diagnostic test accuracy of screening tools for the detection of neurocognitive disorders in older adults post-trauma: A protocol for a systematic review’. https://doi.org/10.5281/zenodo.10978882 ^26^.
