# Supplementary material for: Diagnostic test accuracy of screening tools for the detection of neurocognitive disorders in older adults post-trauma: A protocol for a systematic review
Source: HRB Open Res. 2024 Sep 3;7:33. Originally published 2024 Jun 4. [Version 2] doi: 10.12688/hrbopenres.13894.2 (PMC11437317; doi:10.12688/hrbopenres.13894.2)
Supplement: Peer review history

# Reviewer report: Reviewer response for version 2

**DOI:** 10.21956/hrbopenres.15335.r42249

**Published:** 2024-09-27

**Authors:** Carole Fogg

**Affiliations:** 1 University of Southampton, Southampton, England, UK

Many thanks for the helpful responses. I have no further comments and happy to approve.

Is the study design appropriate for the research question?

Yes

Is the rationale for, and objectives of, the study clearly described?

Yes

Are sufficient details of the methods provided to allow replication by others?

Yes

Are the datasets clearly presented in a useable and accessible format?

Not applicable

Reviewer Expertise:

Older adults health and care, epidemiology.

I confirm that I have read this submission and believe that I have an appropriate level of expertise to confirm that it is of an acceptable scientific standard.

# Reviewer report: Reviewer response for version 2

**DOI:** 10.21956/hrbopenres.15335.r42258

**Published:** 2024-09-25

**Authors:** Joyce Chan

**Affiliations:** 1 School of Public Health and Primary Care, Faculty of Medicine, The Chinese University of Hong Kong, Hong Kong, Hong Kong

This protocol aims to evaluate the performance of screening tools for the identification of NCDs in older persons with acute trauma. My comments are as follows:

 In the Introduction, the authors may consider adding relevant studies or findings about the current assessment tools, or the relevant systematic reviews related to this topic.

 In the methods, I suggest separating the presentation of "cognitive impairment" and "dementia".  It is because the definition and diagnosis of "cognitive impairment" and "dementia" should be different.

Is the study design appropriate for the research question?

Yes

Is the rationale for, and objectives of, the study clearly described?

Yes

Are sufficient details of the methods provided to allow replication by others?

Yes

Are the datasets clearly presented in a useable and accessible format?

Not applicable

Reviewer Expertise:

Research Interests:mild cognitive impairment, dementia, depression, cogntive assessment, psychosocial interventions for older adults

I confirm that I have read this submission and believe that I have an appropriate level of expertise to confirm that it is of an acceptable scientific standard, however I have significant reservations, as outlined above.

# Reviewer report: Reviewer response for version 1

**DOI:** 10.21956/hrbopenres.15237.r40571

**Published:** 2024-07-05

**Authors:** Carole Fogg

**Affiliations:** 1 University of Southampton, Southampton, England, UK

This systematic review protocol provides a clear overview of the study methods and background to the study.

 My main query is about the grouping of “cognitive impairment/dementia” – from the perspective that screening tools which assess cognitive function may then trigger a series of further assessments which may result in a diagnosis of dementia. It is unclear from the way in which this is grouped (if indeed that is the intention) as to what the ‘outcome’ in the case of such screening tools is – will there be a comparison for those screening for cognitive impairment vs clinically confirmed diagnosis of cognitive impairment, and a separate comparison for a screening tool for cognitive impairment which has led to a diagnosis of dementia? If the authors could please clarify what they had intended by combining these, this would be helpful.

 Related to this, the exclusion criteria of ‘studies conducted in environments other than acute care’ may be difficult to apply in situations where although screening occurs in acute care, subsequent follow-up/assessments take place in another environment. The authors could consider altering this to something like ‘studies in which participants are identified in environments other than acute care’, which would allow collection of diagnostic data across settings.

Is the study design appropriate for the research question?

Yes

Is the rationale for, and objectives of, the study clearly described?

Yes

Are sufficient details of the methods provided to allow replication by others?

Yes

Are the datasets clearly presented in a useable and accessible format?

Not applicable

Reviewer Expertise:

Older adults health and care, epidemiology.

I confirm that I have read this submission and believe that I have an appropriate level of expertise to confirm that it is of an acceptable scientific standard, however I have significant reservations, as outlined above.

# Response

**Published:** 2024-08-29

**Authors:** Niamh A. Merriman

**Affiliations:** University College Dublin, Dublin, Ireland

We thank the reviewer for the constructive feedback on this protocol. We now address each point in responses below the review comment.

*This systematic review protocol provides a clear overview of the study methods and background to the study.*

- **Response**: Thank you for reviewing this protocol. We appreciate the feedback on clarity of the description of the methodology and study background.

*My main query is about the grouping of “cognitive impairment/dementia” – from the perspective that screening tools which assess cognitive function may then trigger a series of further assessments which may result in a diagnosis of dementia. It is unclear from the way in which this is grouped (if indeed that is the intention) as to what the ‘outcome’ in the case of such screening tools is – will there be a comparison for those screening for cognitive impairment vs clinically confirmed diagnosis of cognitive impairment, and a separate comparison for a screening tool for cognitive impairment which has led to a diagnosis of dementia? If the authors could please clarify what they had intended by combining these, this would be helpful.*

- **Response**: Thank you for this comment. The condition of interest in this instance is a contemporaneous diagnosis of cognitive impairment **or** dementia. Similar to previous reviews ( https://doi.org/10.1177/23969873211042192), it is not our intention to conduct separate comparisons based on whether the reference standard is a clinical dementia diagnosis or clinical diagnosis of cognitive impairment. We will however conduct a sub-group analysis based on the type of reference standard used (i.e., standardised diagnostic criteria or a validated tool).

*Related to this, the exclusion criteria of ‘studies conducted in environments other than acute care’ may be difficult to apply in situations where although screening occurs in acute care, subsequent follow-up/assessments take place in another environment. The authors could consider altering this to something like ‘studies in which participants are identified in environments other than acute care’, which would allow collection of diagnostic data across settings.*

- **Response**: We agree that a contemporaneous diagnosis of cognitive impairment or dementia is likely to occur in a setting other than acute. We have rephrased the study selection criterion to: *“Studies in which participants are identified in environments other than acute care”*

# Reviewer report: Reviewer response for version 1

**DOI:** 10.21956/hrbopenres.15237.r40568

**Published:** 2024-06-28

**Authors:** Parichita Choudhury, Andrew Ho

**Affiliations:** 1 Banner Sun Health Research Institute, Sun City, AZ, USA

This study aims to validate the accuracy of different screening tools in detecting different neurocognitive disorders in the setting of an acute traumatic event. They wish to do a systematic review utilizing well-established systems to assess the accuracy of studies such as PRISMA-DTA. The inclusion and exclusion criteria are clear and concise to achieve the desired search outcomes. The study utilizes the recommended minimum of two reviewers with a third to act as a tiebreaker to ensure reproducibility, and to present the dataset in 2x2 table to define specificity and sensitivity of each tool is a usable and accessible format. 

 One of the primary concerns lies within the study question itself. 

 The diagnosis of dementia or other cognitive impairment in acute setting is challenging and recommended against given confounding factors of delirium and other medical comorbidities. There are some tools for detecting delirium, but to my knowledge, there are no good tools to diagnose or detect mild cognitive impairment or dementia in an acute clinical environment. Accurate diagnosis of these latter two conditions require testing under nonacute conditions which would require evaluation prior to hospitalization or a trusted care partner at bedside. An initial diagnosis within an acute setting invites significant heterogeneity of conflicting differential diagnoses, and authors should be mindful of this as they conduct this review. This would make diagnostic accuracy of all tools poorer. Just because there is a good question does not always ensure that there will be a good answer.

 Other considerations:

 1. Would the tools included be: patient reported? administered by any healthcare professional? administered by professionals with specific training? 

 2. If reports of any language is included, please be mindful of dearth of validation of screening tools in other languages and cultures. 

 3. Gold standards between studies will vary considerably, and therefore diagnostic accuracy may also have high variability.

 4. The authors should consider separating out head trauma vs. no head trauma data as this may significantly impact the accuracy of results - detection of delirium. 

 The scientific question is an important one and if there are no good tests, then perhaps an answer of "no good tests" may encourage the development of a new tool, which is worthwhile in itself.

Is the study design appropriate for the research question?

Yes

Is the rationale for, and objectives of, the study clearly described?

Partly

Are sufficient details of the methods provided to allow replication by others?

Yes

Are the datasets clearly presented in a useable and accessible format?

Not applicable

Reviewer Expertise:

Dementia

We confirm that we have read this submission and believe that we have an appropriate level of expertise to confirm that it is of an acceptable scientific standard, however we have significant reservations, as outlined above.

# Response

**Published:** 2024-08-29

**Authors:** Niamh A. Merriman

**Affiliations:** University College Dublin, Dublin, Ireland

We thank the reviewer for the constructive feedback on this protocol. We now address each point in responses below the review comment.

*This study aims to validate the accuracy of different screening tools in detecting different neurocognitive disorders in the setting of an acute traumatic event. They wish to do a systematic review utilizing well-established systems to assess the accuracy of studies such as PRISMA-DTA. The inclusion and exclusion criteria are clear and concise to achieve the desired search outcomes. The study utilizes the recommended minimum of two reviewers with a third to act as a tiebreaker to ensure reproducibility, and to present the dataset in 2x2 table to define specificity and sensitivity of each tool is a usable and accessible format.*

- **Response**: Thank you for reviewing this protocol. We appreciate the feedback on appropriateness of the study methodology.

*One of the primary concerns lies within the study question itself.*

*The diagnosis of dementia or other cognitive impairment in acute setting is challenging and recommended against given confounding factors of delirium and other medical comorbidities. There are some tools for detecting delirium, but to my knowledge, there are no good tools to diagnose or detect mild cognitive impairment or dementia in an acute clinical environment. Accurate diagnosis of these latter two conditions require testing under nonacute conditions which would require evaluation prior to hospitalization or a trusted care partner at bedside. An initial diagnosis within an acute setting invites significant heterogeneity of conflicting differential diagnoses, and authors should be mindful of this as they conduct this review. This would make diagnostic accuracy of all tools poorer. Just because there is a good question does not always ensure that there will be a good answer.*

- **Response**: While screening may occur in the acute setting, we agree that a contemporaneous diagnosis of cognitive impairment or dementia is likely to occur in a setting other than acute. We have rephrased the study selection criterion to reflect this. *“Studies in which participants are identified in environments other than acute care.”*

*Other considerations:*

*1. Would the tools included be: patient reported? administered by any healthcare professional? administered by professionals with specific training?*

- **Response: **Thank you for this comment. There is no restriction on tools based on who administers them. Tools administered by any healthcare professional and tools for self-reporting of cognitive function are eligible for inclusion. We have rephrased the study selection criterion to: *“The study aims to validate a screening tool for detection of 1) delirium or 2) cognitive impairment, or dementia at any timepoint in acute care. Tools can be self-report or administered by any healthcare professional.”*

*2. If reports of any language is included, please be mindful of dearth of validation of screening tools in other languages and cultures.*

- **Response: **Thank you for this comment. Studies reported in a language other than English will not be excluded. Our search strategy is comprehensive and will be translated across five databases to ensure maximum identification of relevant studies.

*3. Gold standards between studies will vary considerably, and therefore diagnostic accuracy may also have high variability.*

- **Response: **We agree that there may be high variability in diagnostic accuracy as a function of the reference standard used. For that reason, we plan to conduct a sub-group analysis based on the type of reference standard used (i.e., standardised diagnostic criteria or a validated tool).

*4. The authors should consider separating out head trauma vs. no head trauma data as this may significantly impact the accuracy of results - detection of delirium.*

- **Response: **We agree that patients with and without head trauma should be analysed separately. We have rephrased the following sentence in the data synthesis section to: *“Sub-group analyses will be conducted based on study population (i.e., post-hip fracture, post-surgical, head trauma, other physical trauma)”*

*The scientific question is an important one and if there are no good tests, then perhaps an answer of "no good tests" may encourage the development of a new tool, which is worthwhile in itself.*

- **Response: **Thank you for this comment. We appreciate the feedback on the importance of the research question
